# Primate liver tissue substrate in indirect immunofluorescence diagnostics for patients with dermatitis herpetiformis and celiac disease

**DOI:** 10.3389/fimmu.2023.1104360

**Published:** 2023-02-16

**Authors:** Franziska Schauer, Georgia Tasiopoulou, Daniel Schuster, Max Behrens, Sabine Müller, Dimitra Kiritsi

**Affiliations:** ^1^ Department of Dermatology, Medical Center-University of Freiburg, Faculty of Medicine, University of Freiburg, Freiburg, Germany; ^2^ Institute of Medical Biometry and Statistics, Medical Center, University of Freiburg, Faculty of Medicine, University of Freiburg, Freiburg, Germany

**Keywords:** monkey oesophagus, monkey liver, gluten, Duhring, transglutaminase

## Abstract

**Introduction:**

Dermatitis herpetiformis (DH) is a rare autoimmune, polymorphous blistering disorder, characterized by severe itch or burning sensation, which represents the cutaneous manifestation of celiac disease (CD). The current estimation of DH versus CD is around 1:8 and the affected individuals have a genetic predisposition. Pathogenetically, IgA autoantibodies against the epidermal transglutaminase, an essential constituent of the epidermis, cause DH and are reported to develop through cross-reaction with the tissue transglutaminase, with IgA auto-antibodies causing CD. Immunofluorescence techniques allow for a rapid diagnostics of the disease using patient sera. Evaluation of IgA endomysial deposition with indirect immunofluorescence on monkey oesophagus is highly specific, but moderately sensitive, with some operator-dependent variability. Recently, indirect immunofluorescence with monkey liver as a substrate has been proposed as an alternative, well-functioning diagnostic approach with higher sensitivity in CD.

**Methods:**

The objective of our study was to evaluate whether monkey oesophagus or liver tissue shows advantage for diagnostics in patients with DH, compared to CD. To that end, sera of 103 patients with DH (n=16), CD (n=67) and 20 controls ere compared by 4 blinded experienced raters.

**Results:**

For DH, we found a sensitivity of 94.2% for monkey liver (ML) compared to 96.2% in monkey oesophagus (ME), while specificity in ML was superior (91.6% versus 75%) to ME. In CD, ML had a sensitivity of 76.9% (ME 89.1%) and specificity of 98.3% (ME 94.1%).

**Discussion:**

Our data show that ML substrate is well suitable for DH diagnostics.

## Introduction

Dermatitis herpetiformis (DH) is a polymorphic, subepidermal blistering disease with severe itch or burning sensation, considered to represent the cutaneous manifestation of celiac disease (CD) (1). The blistering mostly affects elbows, knees and buttocks (2). CD is a chronic, small-intestinal T-cell-mediated enteropathy, caused by reaction to dietary gluten and presenting with diarrhoea, unexpected weight lost and vague abdominal discomfort (3, 4). The disease typically occurs in genetically predisposed persons (*HLA-DQ2* or- *DQ8*) with frequent onset in childhood, while is has also been described as an immune therapy-related adverse event (5). Not all patients with CD suffer from DH, the current estimations are at 1 to 8 (6). In general, DH is a rare disease diagnosed in 11.2 to 75.3 per 100,000 people in the United States and Europe with an incidence of 0.4 to 3.5 per 100,000 people per year (7, 8). Whereas DH can manifest solely in the skin, most patients have some degree of histologic features of CD in their small bowel, while in most cases the gastroenterological symptoms are minor (8).

Pathogenetically, CD has been linked to development of the following main types of autoantibodies, namely IgA autoantibodies against tissue and epidermal transglutaminases (TG2, TG3), IgA- and IgG- autoantibodies against deaminated gliadine peptides (dGP) and IgA autoantibodies against the endomysium (EMA) (9). TG2 and IgA-EMA autoantibodies account for around 95% in serological screening; dGP IgA/IgG testing was shown to improve accuracy (10). TG2 is expressed in basal keratinocytes, dermal capillaries, as well as blood vessel walls and small bowel, while TG3 is found in epidermis, oesophagus, brain, the eyes and lowly expressed in the small intestine (2, https://www.proteinatlas.org/). TG2 modifies gluten to gliadin in colon (11), while the main function of TG3, the dominant autoantigen in DH, is the maintenance of the cornified envelope integrity (12). It shows homology to TG2 within the enzymatic active domains (13). The current view of the DH pathogenesis is that patients with DH develop autoantibodies against both TG2 and/or solely against TG3, while individuals with CD have mainly autoantibodies against TG2 (13–15). It has been suggested that DH develops as a result of prolonged gluten exposure and an untreated CD, however no data exist on why antibodies against TG2 and TG3 develop in parallel, or if TG3 merely becomes targeted *via* gradual loss of antigen specificity against TG2 in a subset of individuals with CD (14).

The diagnosis of DH is often difficult and prolonged. Studies revealed a mean time of 3 years between development of the rash and diagnosis, which is significantly increased for female patients (16). An important factor affecting this, is the duration time of undiagnosed preceding CD, partly misdiagnosed as irritable bowel disease (16). The DIF is gold standard with a sensitivity of around 94% (17). It shows micro-granular-fibrillar deposition of IgA (IgA- TG3 complexes), most prominently found at the tips of the dermal papillae, within vessels of the dermal papillae and along the dermoepidermal junction zone (DEJ) (2, 13). Other possible findings are isolated reactivity of C3 at DEJ or IgA deposits along the DEJ of the hair follicles (2). The biopsy site is of vital importance, since the pathognomonic IgA deposits are significantly increased in perilesional, non-affected skin (18). Nevertheless, false positivity of DIF can occur in CD patients without DH characteristic skin changes (2). Serological testing has gained an important role in diagnosis of DH, due to the high specificity and easier application (15). Specifically, immunofluorescence assays of antibodies against endomysium on primate, specifically monkey oesophagus (ME) sections represent the gold standard in serological testing for DH, but also CD (19). The reactivity focuses on the connective tissue layers around the smooth muscle fibers of the lamina muscularis mucosae and tunica muscularis, which highly express TGs. Recently, an immunofluorescence assay with primate liver tissue (monkey liver, ML) was proposed as an alternative, well-functioning diagnostic approach with higher sensitivity in CD (19). On this substrate the antibodies bind on the vessels of the liver, called the sinusoids (Vv. intralobulares) (19). The objective of our study was to evaluate whether ME or ML as substrates show advantage for diagnostics in patients with DH and CD and to gain insights if a correlation to the antibody titers against TG and gliadins exists when employing ME or ML, irrespective of the disease activity.

## Materials and methods

### Serum samples and ethic approval

In total 103 human sera of different patients (DH, n=16, CD, n=67, control=20) were collected between 2008 and 2022 in accordance with the principles of the Declaration of Helsinki and the Ethics Committee University of Freiburg. Blood from residual sera was used, which had no further diagnostic purposes and therefore informed consent was waived, based on Ethics Committee decision (reference no 235/15). In all DH patients diagnosis was made based on clinical picture, histology and IgA deposits on DIF, according to international S2k guidelines (2). Sera collection time point was variable: at initial presentation or at a follow up visit, however some disease activity had still to be present. Sera from the same patient at different time points were not used, in this case the serum from the initial presentation was analysed. Control sera were collected from patients with pruritic skin disorders. Data on age, sex and comorbidities, like diabetes mellitus type I or irritable bowel disease were not collected. The dietary status and specifically the gluten free intake had not been recorded for all patients and were thus not used in the evaluation.

### Raters

Four raters with experience of at least 4 to 10 years in immunofluorescence diagnostics participated in this study and evaluated slides with ME and ML substrates in a blinded manner. Both tests were implemented to the routine already at the time of investigation. The individual results were correlated to serological data of celiac specific autoantibodies (anti-TG2 IgA, dGP IgG and dGP IgA) and defined as true positive (TP), true negative (TN) or false positive (FP) and false negative (FN) per rater.

### ELISA

The levels of serum IgA autoantibodies against TG2 were assessed with anti-TG2 ELiA (Thermo Science, 14-5517-01) with manufacturer’s cut off value of > 10 U/ml. Cut off values of anti-dGP IgG and IgA are at > 10 U/ml (Thermo Science, ELiA GliadinDP IgG, 14-5538-01; ELiA GliadinDP IgA, 14-5539-01). All measurements were made using the programmable ELISA reader Phadia™ 250.

### Indirect immunofluorescence

Cryostat sections for ME and ML were processed as described in manufacturer’s manual (ME Inova; FC 1914-1005; ML Euroimmune, Lübeck, Germany). Immunofluorescence patterns were evaluated with Nikon Eclipse 80i microscope. For visualization, pictures were taken with the imaging software NIS-Element.

### Statistics

Descriptive statistics was performed using Excel. Further, inductive statistical analyses were performed using R (R version 4.1.3) to compute sensitivity, specificity, positive predictive value (PPV), and negative predictive value (NPV) of our results. Therefore, 95% confidence intervals (95% CI) for rater diagnostic statistics were computed using a Wilson Score interval. Fleiss’ kappa was used to evaluate the interrater reliability of agreement among these two systems. Associations in the results between tests were assessed using McNemar’s test for correlated proportions with continuity correction in case of low cell numbers. Level of significance was considered at *P* <.05.

## Results

### Dermatitis herpetiformis

Eight out of 16 DH (50%) sera were positive for anti-TG2 IgA, anti-dGP IgA and IgG autoantibodies. Three out of 16 (19%) patients showed anti-TG2 IgA and anti-dGP IgG positivity and anti-dGP IgA negativity, one serum (6%) was anti-TG2 IgA, anti-dGP IgA positive, one serum showed anti-TG2 IgA reactivity only, whereas three out of 16 (19%) sera were completely negative (see **Table 1**). The mean values of the ELISA analyses used are shown in **Table 2**.

**Table 1:** Number of examined sera used with the respective positive ELISA results for anti-TG2 IgA, anti-dGP IgG and/or anti-dGP IgA, and number of negative sera.

Parameter	DH (%)	CD (%)	Control (%)
No. of sera	16	67	20
TG2 IgA, dGP IgG, dGP IgA	8 (50)	16 (24)	–
TG2 IgA, dGP IgG	3 (19)	3 (4)	–
TG2 IgA, dGP IgA	1 (6)	7 (10)	–
TG2 IgA	1 (6)	14 (21)	–
dGP IgG, dGP IgA	–	4 (6)	–
dGP IgG	–	9 (13)	–
dGP IgA	–	14 (21)	3 (15)
negative	3 (19)	–	17 (85)

CD, celiac disease; DH, dermatitis herpetiformis; TG2, tissue transglutaminase; dGP, deaminated gliadine peptides.

**Table 2:** Mean ELISA levels of examined sera.

Parameter	DH	CD	Control
No. of sera	16	67	20
ELISA Score (U/ml)			
TG2 IgA (mean ± SD)	71.9 ± 52.1	41.9 ± 51.4	0.8 ± 0.9
dGP IgG (mean ± SD)	36.6 ± 43.1	33.8 ± 56.8	0.5 ± 0.3
dGP IgA (mean ± SD)	18.9 ± 18.7	24.7 ± 34.3	3.0 ± 4.8

CD, celiac disease; DH, dermatitis herpetiformis; SD, standard deviation; TG2, tissue transglutaminase; dGP, deaminated gliadine peptides.

### Celiac disease

For the diagnostics 67 sera of different patients with CD were available. Sixteen out of 67 (24%) were positive for anti-TG2 IgA and anti-dGP IgA and IgG; fourteen sera (21%) had anti-TG2 IgA antibodies only; three sera (5%) were positive for anti-TG2 IgA and anti-dGP IgG and seven sera (10%) were positive for anti-TG2 IgA and anti-dGP IgA. Four sera (6%) were positive for anti-dGP IgA and IgG. Fourteen patients (21%) had solely anti-dGP IgA, and 9 patients (13%) had anti-dGP IgG antibodies only. We had no negative sera in our CD cohort (see **Table 1**).

The mean values of the ELISA analyses used are shown in **Table 1**. In general, the anti-TG2 IgA autoantibody titers were significantly higher (*P*=.0392) in the DH group than the CD group, probably because most of them were treatment naïve at sera collection. The titers of the other autoantibodies, however, were similarly distributed within both groups.

### Control sera

Twenty control sera were used from patients with pruritic skin disorders. IIF with the ME and ML substrates showed negative results in all these samples. ELISA results are shown in **Tables 1**, **2**. Note that besides anti-dGP IgA, which were found slightly increased in 3 patients (15%), all other patients (n=17, 85%) had negative ELISA results.

### Rater decisions

In total, 332 decisions have been made for all DH/CD sera. All decisions have been summarized in **Table 3**. For all DH samples the raters had a Fleiss’ kappa of 0.60 in ME and 0.63 in ML, which is a moderate and substantial agreement based on Landis and Koch, respectively (20). The raters’ results revealed a sensitivity of 96.2% for ME compared to 94.2% in ML (*P*=1). In contrast, the specificity was higher for ML evaluation compared to ME (ML: 91,7% versus ME: 75%, *P = .48*), although not significant. Rater decisions on control samples were not taken into consideration for this analysis, since they were all negative.

**Table 3:** Interrater agreement of 332 decisions; calculation of diagnostic sensitivity, specificity and predictive values of ME and ML, in relation to all ELISA results.

Parameter	DH		*P*	CD		*P*
	ME	ML		ME	ML	
Fleiss’ kappa	0.60	0.63		0.73	0.72	
TP	50	49		139	120	
TN	9	11		106	110	
FP	3	1		6	2	
FN	2	3		17	36	
Sensitivity (95%CI)	0.962 (0.87 - 0.989)	0.942 (0.844 - 0.98)	1	0.891 (0.832 - 0.931)	0.769 (0.697 - 0.828)	<.00
Specificity (95%CI)	0.75 (0.468 - 0.911)	0.917 (0.646 - 0.985)	0.48	0.946 (0.888 - 0.975)	0.982 (0.937 - 0.995)	0.29
PPV (95%CI)	0.943 (0.846 - 0.981)	0.98 (0.895 - 0.996)		0.959 (0.913 - 0.981)	0.984 (0.942 - 0.995)	
NPV (95%CI)	0.818 (0.523 - 0.949)	0.786 (0.524 - 0.924)		0.862 (0.79 - 0.912)	0.753 (0.678 - 0.816)	

CD, celiac disease; CI, confidence interval; DH, dermatitis herpetiformis; TN, true negative; TP, true positive; FN, false negative; FP, false positive; PPN, positive predictive value; NPV, negative predictive value; ME, monkey oesophagus; ML, monkey liver.

*Level of significance *P* <.05.

**Figure 1:**
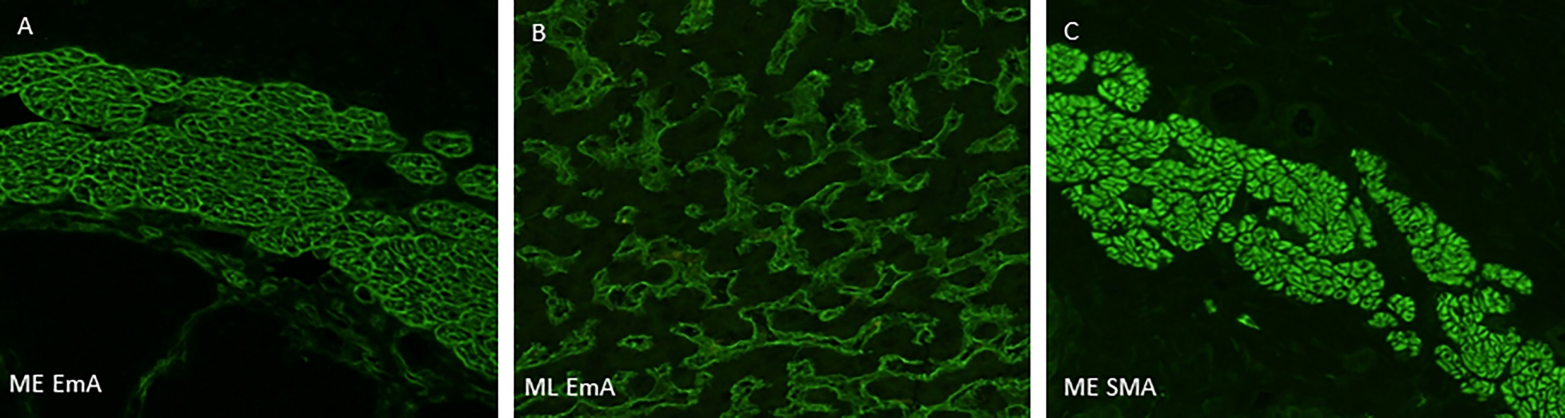
Representative staining pattern of the indirect immunofluorescence-based primate monkey oesophagus and monkey liver assays. **(A)** Serum of a patient with suspected DH shows IgA autoantibodies with typical honeycomb-like, endomysial staining on monkey oesophagus section. **(B)** Serum of the same patients shows endomysial autoantibodies within the liver sinusoids (Vv. intralobulares). **(C)** Serum of a patient with only anti-SMA antibodies shows staining of smooth muscle fibres. *DH, dermatitis herpetiformis; CD, celiac disease; SMA, smooth muscle antibodies; ME, monkey oesophagus; ML, monkey liver*.

Interestingly, one serum that was rated positive by some raters showed a different pattern than the characteristic honeycomb-like, endomysial staining in the ME sections, this patient has been excluded from the further analysis (**Figures 1A–C**). This hints one limitation of the ME substrate, namely that SMA autoantibodies might easily be misinterpreted as EMA antibodies. The diagnostic sensitivity and specificity of ML and ME, as well as positive and negative predictive values for the analyses, are shown in **Table 3**.

### Univariate anti-TG2 IgA positivity

When considering only anti-TG2 IgA positivity, our analysis showed that ML has generally fewer false positive results (**Table 4**). It shows that the specificity is significantly higher for ML compared to ME (ME: 0.812 (95% CI 0.57 - 0.934), ML: 1.0 (95% CI 0.806 - 1.0), *P*=.00). Further, Fleiss’ kappa was 0.32 and 0.51 for ME and ML, respectively. This indicates a difference regarding rater accordance within the anti-TG2 IgA positivity subset. As a limitation, sera of DH were not considered separately, since sample size was only one serum. Thus, the main data were based on sera of CD. Generally, when assessing our data based only on anti-TG2 positivity, it appears that sensitivity is lower compared to the whole data set. Thus, there is a difference regarding the rater diagnostic metrics for the subset of positive IgA titers compared to all probes.

**Table 4:** Calculations of diagnostic sensitivity, specificity and predictive values of ME and ML in relation to subset of anti-TG2 IgA positive sera. CD and DH combined, since DH has only 2 samples.

Parameter	All (DH and CD)		*P*
	ME	ML	
Fleiss kappa	0.32	0.51	
TP	34	28	
TN	13	16	
FP	3	0	
FN	10	16	
Sensitivity (95%CI)	0.773 (0.63 - 0.872)	0.636 (0.489 - 0.762)	0.11
Specificity (95%CI)	0.812 (0.57 - 0.934)	1.0 (0.806 - 1.0)	*>.00
PPV (95%CI)	0.919 (0.787 - 0.972)	1.0 (0.879 - 1.0)	*>.00
NPV (95%CI)	0.565 (0.368 - 0.744)	0.5 (0.336 - 0.664)	1

CD, celiac disease; CI, confidence interval; DH, dermatitis herpetiformis; TN, true negative; TP, true positive; FN, false negative; FP, false positive; PPN, positive predictive value; NPV, negative predictive value; ME, monkey oesophagus; ML, monkey liver.

*Level of significance *P* <.05.

### Positivity of anti-TG2 IgA combined with positive dGP IgG or positive dGP IgA

The subset analysis for probes with TG2 IgA positive and positive dGP IgG or positive dGP IgA are presented in **Table 5**. Sensitivity is generally a bit higher for this subset. Specificity cannot be computed, since all sera in the subset were truly positive. For CD, the sensitivity of ME is worse compared to ML (ME: 83.7% (95% CI 0.754 - 0.895), ML: 91.3% (95% CI 0.844 - 0.954), *P=*.06*)*. For DH, the sensitivity of ME is significantly higher, although based on a small sample size. The Fleiss’ kappa values are marginally higher for ML compared to ME, although both with a moderate accordance based on (20) with values of 0.51 and 0.45, respectively (20).

**Table 5:** Calculations of diagnostic sensitivity, specificity and predictive values of ME and ML in relation to subset of anti-TG2 IgA positive sera combined with either a positive anti-dGP IgG or a positive anti-dGP IgA.

Parameter	DH		*P*	CD		*P*
	ME	ML		ME	ML	
Fleiss’ kappa	–	–		0.45	0.51	
TP	48	46		95	87	
TN	0	0		0	0	
FP	0	0		0	0	
FN	0	2		9	17	
Sensitivity (95%CI)	1.0 (0.926 - 1.0)	0.958 (0.86 -0.988)	*<.00	0.837 (0.754 - 0.895)	0.913 (0.844 - 0.954)	.06
Specificity (95%CI)	–	–		–	–	
PPV (95%CI)	1.0 (0.926 - 1.0)	1.0 (0.923 - 1.0)	1	1.0 (0.961 - 1.0)	1.0 (0.958 - 1.0)	1
NPV (95%CI)	–	0.0 (0.0 - 0.658)		0.0 (0.0 - 0.299)	0.0 (0.0 - 0.184)	1

CD, celiac disease; CI, confidence interval; DH, dermatitis herpetiformis; TN, true negative; TP, true positive; FN, false negative; FP, false positive; PPN, positive predictive value; NPV, negative predictive value; ME, monkey oesophagus; ML, monkey liver.

*Level of significance *P* <.05.

## Discussion

IgA-based indirect immunofluorescence and enzyme linked immune sorbent assays (ELISA) are the main serological methods for DH diagnostics. The microscopic detection of EMA in the sera of the patients is a semi-quantitative analysis. However, experienced diagnosticians are needed for a high quality evaluation to avoid false negative results (21). Current guidelines recommend using cryosections from ME for indirect IF (IIF) diagnostics, although other substrates such as human umbilical cord, human appendix, monkey uterus or rabbit oesophagus may also be considered as smooth muscle fibre substrates (2). The sensitivity is described between 60-90%, with specificity of up to 100% in untreated DH cases (10, 22).

We here used primate ML tissue as substrate for IIF detection of EMA antibodies to evaluate whether by its use we can increase sensitivity of EMA IIF diagnostics. IgA EMA antibodies on ML have been reported to represent the reticulin antibody binding pattern in CD (23). Reticulin antibodies were among the first antibodies described in CD in the early 1970s and were reported to have excellent specificity, but poor sensitivity. Being found in only 40-60% of cases of active CD, the test method was displaced (24). In an unselected, small DH cohort IgA reticulin autoantibodies detected by IIF with ML were present in 10-40% of the patients with increasing incidence corresponding to the severity of the jejunal abnormalities (23, 25, 26).

We compared ML and ME diagnostics in both DH and CD patients in a blinded manner and thereafter categorized them, based on their antibody profile. In our DH cohort, 75% (12/16) of the sera were positive in ML IIF. In CD an inaccordance (FN) in eight out of 67 sera (12%) was detected, interestingly in patients with anti-TG2 IgA below 20 U/ml. Considering that levels of TG2 autoantibodies can be affected by diet status, a limitation of the study is the lack of knowledge on the gluten intake of our patients. In a retrospective pediatric study a decrease of around 70% in the anti-TG2 levels within 3 months of a gluten-free diet was shown, while around 80% of the children were sero-negative for anti-TG2 and in the IIF diagnostics only after 2 years of the diet (27). Twenty-seven sera of the CD cohort (40%) showed antibodies against anti-dGP IgA and IgG in combination with anti-dGP IgA or anti-dGP IgG antibodies only and were taken at different time points during clinical course. An isolated positive anti-dGP IgG test in absence of anti-TG2 IgA antibodies at initial diagnostics may be nonspecific and often FP in infancy. It therefore has no predictive value for CD (28). Isolated searching for IgA antibodies against dGP also has low specificity and is not recommended in childhood (29). Although the dietary status of our patients is unknown, all had some signs of disease activity or persistence, when the sera were taken.

Our initial data indicate that ML IIF is useful as primary or confirmatory assays in DH with positive anti-TG2 IgA at initial diagnosis, but might be less suitable as a follow-up parameter. The results also indicate an equivalence for ML and ME, as no statistically significant differences were found. Due to low sample size, confidence intervals for DH are quite wide. Both sensitivity and specificity seem to perform slightly better for ML in DH. The bigger sample size for CD leads to a narrower confidence interval and a better interpretability of results. Regarding sensitivity ME seems to be slightly better, which is statistically significant (P <.00). Regarding specificity ML performed slightly better, but not significantly. Further analyses in larger DH cohorts would be desirable to confirm our results.

In our cohort we did not test for TG3 autoantibodies, which are reported to be the autoantigen for DH, this is a limitation that should be addressed in future studies. In future, additional immunoblot (IB) testing with bianalyte detection of IgA against TG2 and nanopeptides of gliadin could be of interest. In a recently published cohort, IB showed 78% sensitivity, 100% specificity, 100% positive predictive value, and 82% negative predictive value in relation to ELISA against TG2 alone. Henceforth, a multiplex approach for DH diagnostics with multianalyte IIF and multivariant ELISA profile will probably replace monoparametric diagnostics (30).

## Conclusion

The results show that ML substrate is suitable for EMA diagnostics in DH. It appears to be slightly easier for the raters to evaluate and might be interesting for inexperienced raters, since misinterpretation occurring in ME diagnostics can be avoided. The study is limited by its retrospective approach and the fact that sera were taken at different time points considering diet status and disease activity. Also, data on anti-TG3 IgA ELISA diagnostics in DH are not available and were not considered. Since DH and CD are rare disorders and we here present a single-centre study, statistical significance of the data was difficult to achieve in this cohort.

## Data availability statement

The original contributions presented in the study are included in the article/supplementary material. Further inquiries can be directed to the corresponding author.

## Ethics statement

The studies involving human participants were reviewed and approved by Human Ethics Committee University of Freiburg (reference No.235/15). Written informed consent for participation was not required for this study in accordance with the national legislation and the institutional requirements.

## Author contributions

The idea for this research topic was brought up by FS and DK, who also performed data acquisition and sorting. FS, DK, GT and DS were blinded evaluators of the stainings. MB performed statistical analysis. SM provided patient sera and revised the manuscript. The manuscript was drafted and designed by FS and DK. All authors contributed to the article and approved the submitted version.
